# Preparation of Water-Soluble Acetylaminoglucan with Low Molecular Weight and Its Anti-Tumor Activity on H22 Tumor-Bearing Mice

**DOI:** 10.3390/molecules27217273

**Published:** 2022-10-26

**Authors:** Jinfang Zhang, Keyao Dai, Mei Li

**Affiliations:** 1 Key Laboratory of Industrial Fermentation Microbiology Ministry of Education & Tianjin Key Laboratory of Industrial Microbiology, The College of Biotechnology, Tianjin University of Science and Technology, Tianjin 300457, China; 2 State Key Laboratory of Food Nutrition and Safety Ministry of Education, College of Food Engineering and Biotechnology, Tianjin University of Science and Technology, Tianjin 300457, China; 3 College of Life Sciences, Yantai University, Yantai 264005, China

**Keywords:** low molecular weight acetylaminoglucan, preparation, anti-tumor activity

## Abstract

In this study, a novel low molecular weight of acetylaminoglucan (AGA) was obtained and its antitumor activity on H22 tumor-bearing mice was investigated. The results of UV, HPLC and FT-IR showed that AGA present high purity with low molecular weight of 2.76 × 10^3^ Da. Animal experiments showed that AGA could inhibit the proliferation of tumor cells in H22 tumor-bearing mice by protecting the immune organs, enhancing the phagocytosis ability of macrophages, killing activity of NK cells and proliferation capacity of lymphocytes, improving the levels of cytokines in vivo and regulating the distribution of lymphocyte subsets, and the tumor inhibition rate reached to 52.74% (50 mg/kg). Cell cycle determination further indicated that AGA could induce apoptosis of tumor cells and arrests it in S phase. These results will provide a data basis for the potential application of AGA in pharmaceutical industry.

## 1. Introduction

Cancer is one of the most common malignant tumors with complex pathogenesis, and its common symptoms are localized with lumps, ulcers and bleeding, etc. Most malignant tumors do not have obvious symptoms in the early stage, resulting in a late stage when the disease occurs, which undoubtedly increases the difficulty of treatment [1,2]. Surgery, chemotherapy and radiotherapy are common methods for treating tumor diseases, but their therapeutic effects have significant limitations due to the host’s tendency to develop resistance to chemotherapy drugs [3]. Therefore, the search for novel natural antitumor products has begun in recent years [4].

Polysaccharides are a class of carbohydrate with special biological activities such as immunomodulation, antiviral, antitumor and hypoglycemia, which exists widely in nature [5,6]. In general, small molecules polysaccharides are easier to digest than large ones and are therefore more potent to kill tumor cells in vivo. Chitin is a polysaccharide abundant in nature and is the second largest natural macromolecule, which has a variety of biological activities, such as non-toxic antibacterial, antitumor, lipid-lowering, enhancing immunity and so on, which is widely used in medical and drug development fields because of its excellent biocompatibility [7].

Acetylaminoglucan is a poorly water-soluble polysaccharide, compared to chitin, which has better water solubility but much lower activity after deacetylation [8,9,10]. The industrial production of acetylaminoglucan is usually by chemical and enzymatic methods. Chemical preparation of acetylaminoglucan is the most commonly used preparation method in the industrial production of acetylaminoglucan, which is simple and efficient to operate, but this method has a serious environmental pollution problem and a destructive effect to the ecosystem [11]. In contrast, the hydrolytic enzymes required for the enzymatic preparation of acetylaminoglucan are more difficult to obtain and the conditions required for the reaction are not easily controlled, resulting in high production costs. Therefore, it is crucial to seek novel methods for the preparation of acetylaminoglucan.

In this study, a water-soluble small molecule acetylaminoglucan (AGA) was extracted from crude chitin. H22 hepatocellular cells were used to establish a H22 tumor-bearing mice model, and cyclophosphamide (CTX, 30 mg/kg) was used as a positive control to investigate the antitumor activity of different doses of AGA, these results would provide a theoretical basis for the clinical application of acetylaminoglucan.

## 2. Materials and Methods

### 2.1. Materials

Murine H22 hepatocarcinoma cells were purchased from Shanghai Institute of Biological Sciences (Chinese Academy of Sciences, Shanghai). Concanavalin A (ConA), RPMI-1640 medium, cyclophosphamide (CTX), Lipopolysaccharide (LPS), fetal bovine serum, propidium iodide (PI), FITC anti-mouse CD19, FITC anti-mouse CD8, PE anti-mouse CD4, PE antimouse CD3 antibodies, cytokine detecting ELISA kits and DNA content quantitation kits were purchased from Solarbio Technology Co., Ltd. (Beijing, China). Tumor necrosis factor -α (TNF-α), interleukin-2 (IL-2), interferon -γ (IFN-γ), and interleukin-4 (IL-4) detection kits were purchased from Nanjing Jiancheng Institute of Biological Engineering (Nanjing, China). Dimethyl sulfoxide (DMSO) and crude chitin were obtained from Jiangtian Chemical Technology Co., Ltd. (Tianjin, China).

### 2.2. Extraction and Purity Analysis of AGA

First, 100 g of crude chitin was dissolved in concentrated hydrochloric acid solution (90 °C, 3 h), and then the final concentration reached 80% by ethanol precipitation. After being stationary and centrifuged, the alcohol-soluble components were collected and then purified by Sephadex G-25 to obtain acetylaminoglucan (AGA). AGA (1 mg/mL) was scanned in a range of 200 to 800 nm using a Synergy HTX microplate reader (BioTek, Winooski, VT, USA) in steps of 2. The molecular weight of AGA was determined by Agilent 1200 high performance liquid chromatography on TSK-GEL G4000PW XL (7.8 mm × 300 mm) column with an RI detector of 35 °C and a column temperature of 30 °C. The sample concentration was 1 mg/mL, and the AGA sample was filtered by 0.45 μm aqueous needle filter membrane before loading, and the injection volume was 20 μL, the flow rate was 0.8 mL/min, and ultrapure water was used as the mobile phase [12]. The relative molecular weight of AGA was calculated by using the peak time and the logarithm of molecular weight of glucose standard substances T1, T3, T10, T40, T70, T110 and T1000 as the standard curve. KBr (150 mg) and AGA (1 mg) samples were weighed and ground to the wall and then pressed. After that, Fourier infrared detection was performed in the wavelength range of 400–4000 cm^−1^ [13].

### 2.3. Design of the Animal Model

BALB/C mice (female, 18–22 g) were purchased from SPF Beijing Biotechnology Co., Ltd., and reared under standard constant temperature (20–25 °C) and relative humidity (45–55%) with light/dark cycles for 12 h. All animal experiments were carried out in accordance with Laboratory Animal Care protection principles and were ratified with the Local Ethics Committee for Animal Care and Use at Tianjin University of Science and Technology [14].

After feeding for one week to adapt to the environment, these mice were randomly divided into 5 groups (*n* = 10 mice), including blank group, model group, positive group (CTX, 30 mg/kg), AGA group (25 mg/kg) and AGA group (50 mg/kg) [15]. Blank and model group were treated with 0.2 mL sterile normal saline, and dose groups was treated with 25 mg/kg and 50 mg/kg AGA by intragastric administration, respectively. After 15 days of continuous intragastric administration, 2.5 × 10^6^ H22 hepatocarcinoma cells (each mouse) were subcutaneously inoculated in the right forelimb of mice (except the blank group). Cyclophosphamide (CTX), as a chemotherapy drug for the treatment of a variety of cancer diseases, could effectively inhibit tumor growth and was usually used orally or intravenously [16]. Therefore, CTX was used as a positive control in this study, and mice were injected intraperitoneally with 30 mg/kg CTX daily after tumor induction [17], and mice in the dose group were given intragastric administration for another 15 days [18].

### 2.4. Immune Organs Indices and Inhibitory Ratio

All experimental animals were kept by the five principles of freedom of welfare and the “3Rs”. After H22 cell inoculation (16th day), the mice were euthanized by the cervical dislocation method based on humanitarian principles, all mice were weighed and sacrificed, and blood samples were acquired [19]. Their thymus, spleen and solid tumor organs were immediately removed and weighed. Additionally, the immune organ indices and tumor inhibitory rates were calculated as follows: immune organ indices = A1/A2, where A1 represents thymus/spleen weight and A2 represents mouse body weights, inhibitory rate (%) = (M1 − M2)/M1 × 100%, where M1 and M2 were the average tumor weight of the model group and dose groups, respectively [20].

### 2.5. Blood Routine Examination

Blood routine refers to the examination to judge the health status of blood and some diseases by observing the number and distribution of blood cells in blood, including the examination of white blood cells, red blood cells, and platelet systems. The eyeball blood of mice was collected and anticoagulated by EDTA-K_2_, and then the indexes were detected by automatic hematology analyzer [21].

### 2.6. Assays of Splenic Lymphocyte Proliferation Activity

The phagocytic capacity was detected by sterile neutral red solution according to previous methods. Under sterile conditions, 5 mL normal saline was injected into the abdominal cavity of mice, and then ascites was collected and centrifuged (1000 rpm, 5 min) to acquire macrophages. After 2 h incubation (37 °C, 5% CO_2_), the cells that did not adhere were washed with PBS and then cultured (24 h). Then, neutral red solution (100 μL (0.075%, m/v)) was added to each well (2h), and cell lysate containing acetic acid and anhydrous ethanol (1:1) was added after washing with PBS for three times. The phagocytosis was measured at OD_550_ nm [22].

NK cells were isolated by mouse spleen NK cell isolation kit. NK cells and tumor cells (20:1) were co-cultured on 96-well plates and supplemented (48 h). H22 cells and NK cells were cultured separately as control group, and NK cells activity was detected at OD_570_ nm. NK cells activity (%) = (OD_1_ + OD_2_ − OD_3_)/OD_1_ × 100, where OD_1_, OD_2_, OD_3_ represent the OD values of H22 cells (target cells), NK cells (effector cells) and co-cultured cells, respectively [23]. The proliferation activity of splenic lymphocytes was measured by MTT.

### 2.7. Distribution of Lymphocyte Subsets in the Peripheral Blood

Blood samples from tail tip of mice were collected 500 μL, treated with erythrocyte lysate and divided into two tubes (250 μL/each tube). Corresponding monoclonal antibody (CD19^+^, CD3^+^, CD4^+^ and CD8^+^) was added, and incubated for 30 min in the dark, erythrocytes were removed again, and then detected by flow cytometry (FACSCallibur, BD, USA) [24].

### 2.8. Cytokine Levels Evaluation and Cell Cycle Distribution

Cytokine detecting ELISA kit was used to detect the levels of cytokines (TNF-α, IFN-γ, IL-2 and IL-4) [25]. The cell cycle distribution of H22 solid tumor was detected by previous methods [26].

### 2.9. Statistical Analysis

All data were expressed as the mean ± standard deviation (S.D). Statistical significance was analyzed by *t*-test and one-way analysis of variance (ANOVA) using SPSS version 23.0. The value of *p* < 0.05 was considered to be statistically significant.

## 3. Results and Discussion

### 3.1. UV, HPLC and FT-IR Analysis of AGA

**Figure 1:**
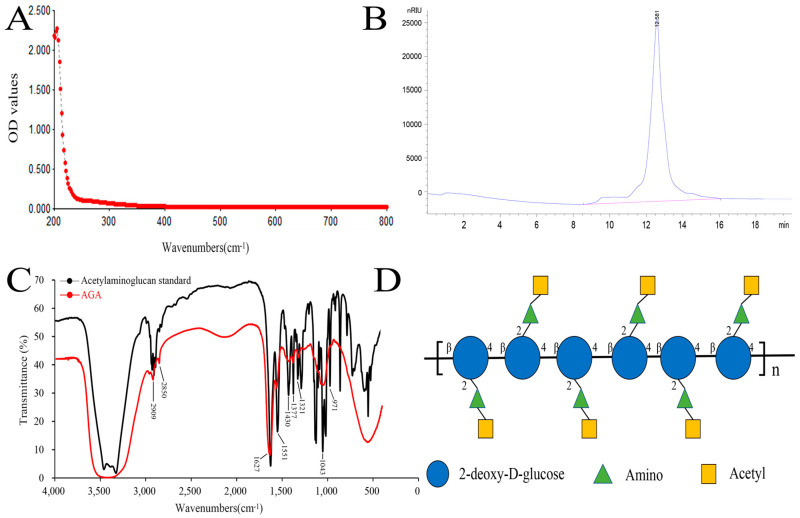
UV spectrum (**A**), HPLC (**B**), FT-IR (**C**) and structure formula (**D**) of AGA.

In this paper, a low molecular weight acetylaminoglucan (AGA) was extracted. We obtained 56 g of AGA by extraction and purification of 100 g of crude chitosan with a yield of 56%. The purity and characteristic functional groups of AGA were detected by UV spectrum analyzer, high performance liquid chromatography and FT-IR detection. A260 nm and A280 nm are the absorption wavelengths of the highest absorption peaks of nucleic acids and proteins, respectively. A smooth curve was observed in the UV spectrum of AGA, with no light absorption at 260 nm and 280 nm, indicating that AGA does not contain nucleic acids and proteins (Figure 1A) [27]. A single peak retention time of 12.582 min was observed from HPLC, showing a single symmetrical narrow peak, indicating that AGA is a small molecular polysaccharide with uniform molecular weight (Figure 1B Regression equation (Rt) was established according to molecular weight (Mw) and corresponding retention time: y = 0.3286x + 9.2747, R^2^ = 0.9990 (y = Ig Mw, x = Rt), the calculated molecular weight of AGA was 2.76 × 10^3^ Da.

Figure 1C showed the FT-IR scan results of acetylaminoglucan standard and AGA. By comparison, at 3420 cm^−1^ were the multiple absorption peaks formed by O-H stretching vibration and -NH stretching vibration absorption peaks [28]. Two stretching vibration absorption peaks of C-H are located at 2909 cm^−1^ and 2850 cm^−1^. The first characteristic absorption peak of acetylaminoglucan was 1627 cm^−1^, the second characteristic absorption peak of acetylaminoglucan was 1551 cm^−1^, and the third characteristic absorption peak of acetylaminoglucan was 1321 cm^−1^ [29]. The absorption peak at 1430 cm^−1^ formed by C-H variable angle vibration. Several absorption peaks between 1400 cm^−1^ and 1200 cm^−1^ are caused by stretching vibration of C-O-C and C-O-H, and 971 cm^−1^ indicates the presence of β-glycosidic bonds in AGA, which are characteristic absorption peaks of polysaccharides. The structural formula of AGA is shown in Figure 1D.

### 3.2. Tumors and Immune Organs Weights

The thymus is an important lymphoid organ in the body, closely related to the body’s immune function, and its main function is to produce T lymphocytes. The spleen removes foreign bodies, bacteria, senescent and dead cells from the blood, has hematopoietic functions, and plays an important role in anti-inflammation. When the body becomes abnormal, the thymus gland begins to atrophy and the spleen develops different abnormalities [30,31].

**Figure 2:**
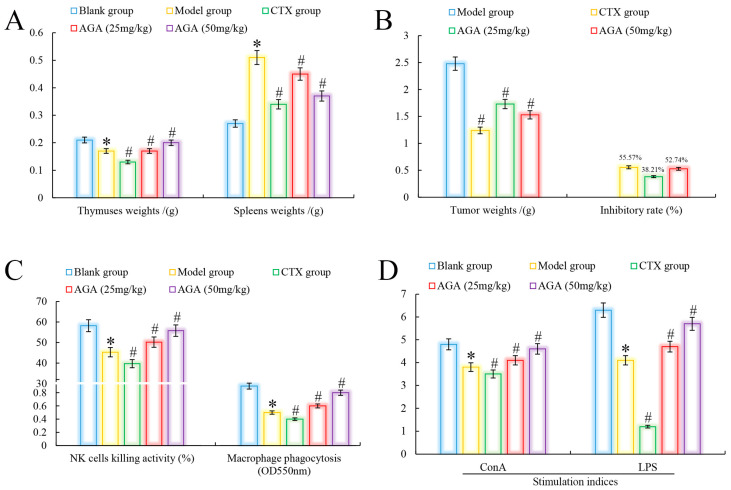
Antitumor effects of AGA in vivo. Immune organ indices (**A**) and tumor inhibitory rate (**B**). NK cell killing activity, macrophage pinocytosis (**C**), and splenic lymphocyte proliferation activities (**D**). * *p* < 0.05, compared with Blank group; ^#^ *p* < 0.05, compared with Model group.

Immune organs indices are an important indicator to evaluate whether AGA has antitumor activity on H22 tumor-bearing mice [32]. As could be seen from Figure 2A, in the model group, the thymus of mice showed atrophy and abnormal splenomegaly (*p* < 0.05) compared with the blank group. In addition, we noted that compared with the blank group, the organs indices (thymus and spleen) of the CTX group indicated that CTX had significant toxic and side effects on immune organs while inhibiting tumor growth in H22 tumor-bearing mice [33]. The thymus indices of mice in dose groups were significantly increased, spleen indices decreased, and in a dose dependent manner (*p* < 0.05) [34]. It was observed that the tumor inhibition rate of CTX on H22 tumor-bearing mice reached to 55.57%, while the tumor inhibition rate of AGA at 25 mg/kg and 50 mg/kg reached to 38.21% and 52.74%, respectively, indicating that AGA has a certain anti-tumor effect (Figure 2B) [35].

Lymphocytes are the core of the body’s immune response and are divided into three categories: T, B and NK natural killer cells. Among them, T cells and B cells are mainly responsible for cellular and humoral immunity, respectively, and their proliferative capacity can be detected by stimulation with ConA and LPS, respectively [36]. NK cells are important immune factors in anti-infection and anti-tumor. Macrophages are the major phagocytes in inflammation and play an important role in wound healing [37]. Compared with the blank group, model group mice abdominal cavity macrophage phagocytosis, NK killer cell activity and lymphocyte proliferation capacity decreased significantly (Figure 2C,D), compared with model group, the phagocytic capacity of peritoneal macrophages, killing activity (NK cells) and proliferation ability of lymphocytes were significantly increased after AGA treatment (*p* < 0.05) and in a dose-dependent manner. At the same time, we observed that these immune abilities were lowest in the CTX group, indicating that CTX has a strong toxic and side effect on the body [38].

### 3.3. Routine Analysis of Blood

**Figure 3:**
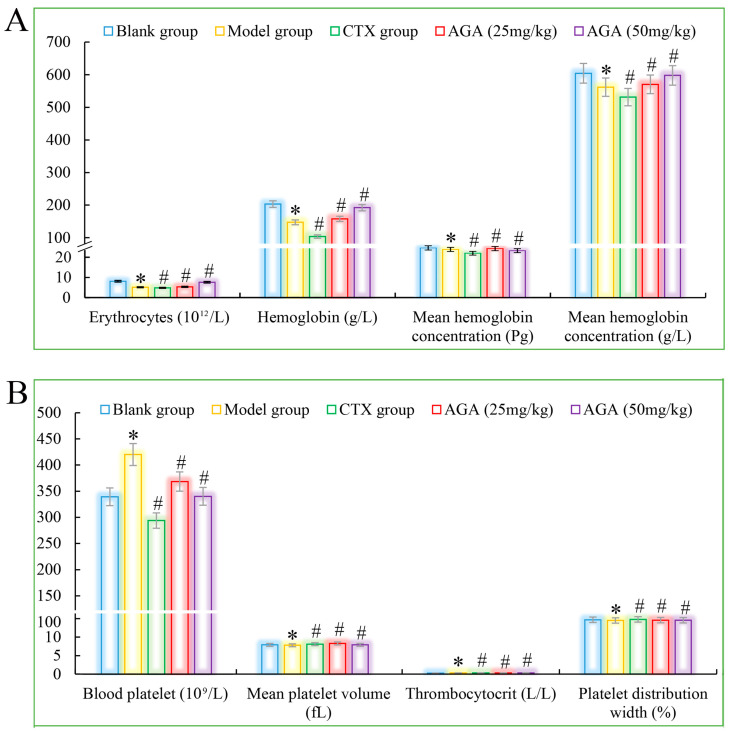
The blood cytokines detections of AGA on H22 tumor bearing mice. (**A**) Erythrocytes, hemoglobin, and proportion of mean hemoglobin. (**B**) Mean platelet volume, number, volume and distribution width. * *p* < 0.05, compared with Blank group; ^#^ *p* < 0.05, compared with Model group.

The routine blood test results are shown in Figure 3A which was mainly includes the erythrocyte system (average number of red blood cells, hemoglobin, red blood cell volume, and average hemoglobin concentration), platelet system (mean platelet volume, platelet count, and platelet distribution width) and leucocyte system (the amount of leukocytes, lymphocytes, intermediate cells, granulocytes and their percentages) [39].

**Figure 4:**
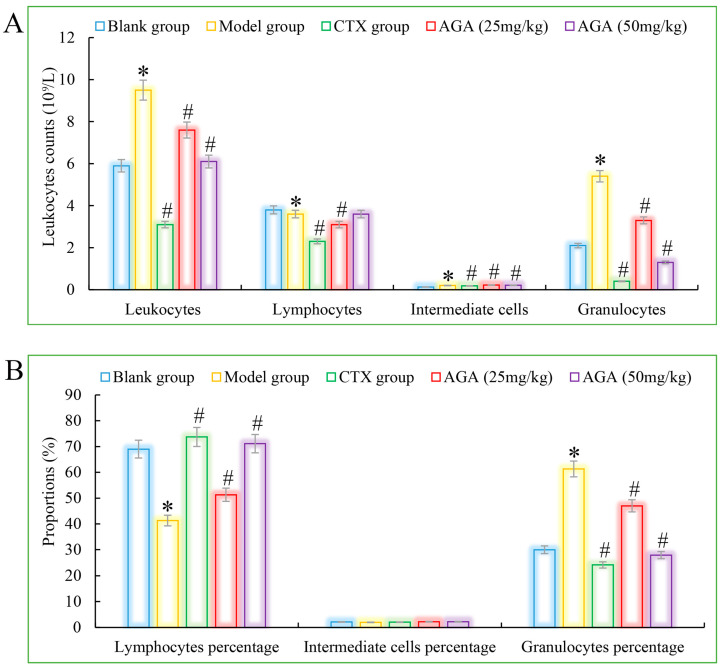
The leucocyte system counts (**A**) and its proportions (**B**) detections of AGA on H22 tumor bearing mice. * *p* < 0.05, compared with Blank group; ^#^ *p* < 0.05, compared with Model group.

Compared with the blank group, the number of erythrocytes, hemoglobin, lymphocyte ratio, lymphocyte ratio, and granulocyte ratio of mice in the model group decreased significantly (Figure 3A and Figure 4A), while the levels of blood platelets, leukocytes, and granulocytes increased significantly (Figure 3B and Figure 4B), which are all distinctive features of malignant tumors, indicating that the growth and proliferation of tumor cells cause anemia, severe tissue damage, and inflammation. Compared with the model group, the number of erythrocytes, hemoglobin dose group mice, lymphocytes, lymphocyte ratio, and granulocyte ratio significantly increased (Figure 3A and Figure 4A), while platelet, leukocyte, and granulocyte levels significantly decreased and were dose correlated (Figure 3B and Figure 4B), indicating that AGA treatment could alleviate malignancy-induced adverse reactions and enhance the immune activity of H22 tumor-bearing mice. The CTX treatment group showed that cyclophosphamide could inhibit the growth of malignant tumors and alleviate tumor-induced adverse effects.

### 3.4. Distributions and Proportions of T Cells Subsets

Peripheral blood lymphocytes are mainly composed of T cells, B cells and lymphocytes. Under normal conditions, the ratio of each lymphatic subpopulation is relatively stable and mutually regulated to maintain normal immune function of the body [40]. When the ratio of different lymphocyte subpopulations changes, the body will suffer from immune dysfunction, which will lead to a series of diseases. Therefore, detection of the distribution of each lymphocyte subpopulation by flow cytometry is an important indicator of immune function [41]. CD3^+^ is mainly expressed by T cells, responsible for cellular immune functions, involved in anti-tumor, etc. The T lymphocyte subpopulation assay consists of total T cells (CD3^+^) and their subpopulations (helper lymphocytes CD4^+^), which play an important role in the body’s anti-tumor, where CD4^+^ cells play an important role in synergistic killing of tumor cells and can cause immune escape of tumor cells when the number of CD4^+^ is reduced. CD19^+^ is the most important marker of B cells and is associated with B cell proliferation differentiation and antibody production, with hematopoietic function [42].

**Figure 5:**
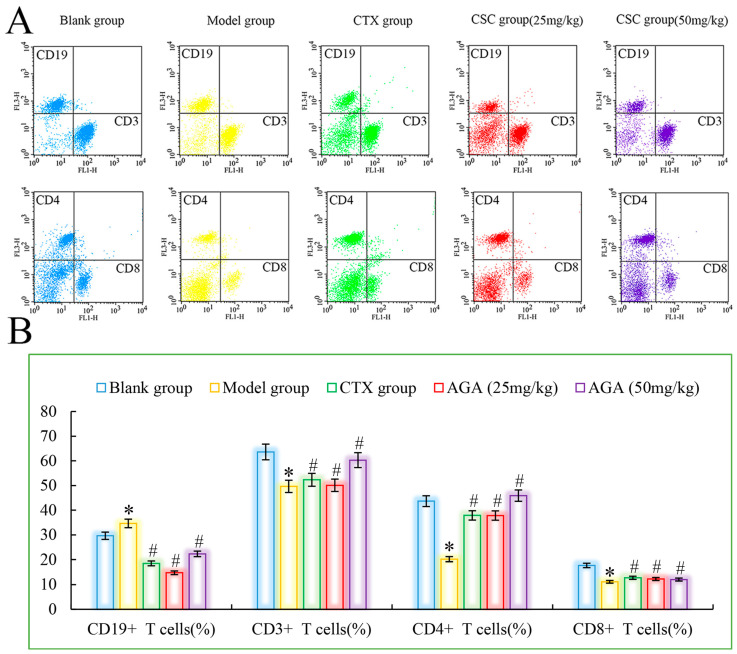
The distribution (**A**) and proportions (**B**) of lymphocytes subsets in peripheral blood. * *p* < 0.05, compared with Blank group; ^#^ *p* < 0.05, compared with Model group.

As could be seen from Figure 5, compared with the blank group, CD3^+^, CD4^+^ and CD8^+^ cells in the model group were significantly reduced, while CD19^+^ was significantly increased (*p* < 0.05), which were all characteristics of T lymphocyte subsets in peripheral blood of malignant tumor, indicating that the immune function of mice in the model group was immunosuppressed. The ability of the host to recognize and kill mutant cells decreases, and tumor growth and metastasis occur. CTX group showed that CTX treatment could inhibit the growth and proliferation of tumor cells in mice [43]. Compared with model group, CD3^+^, CD4^+^ and CD8^+^ cells in dose group were significantly increased, while CD19^+^ was significantly decreased (*p* < 0.05), suggesting that AGA can improve the immune function of H22 tumor-bearing mice, improve the recognition of cytotoxicity in mice, and finally achieve the objective of inhibiting the growth of H22 cells in vivo [44].

### 3.5. Cytokine Levels in Sera

The main function of cytokines is to regulate the immune response of the body, which is related to the hematopoietic function and inflammatory response of the body, with multiple regulatory effects, promoting apoptosis and facilitating the repair of traumatic tissues. The important lymphokines include TNF-α, IL-2 and IFN-γ. TNF-α can kill virus-infected target cells or tumor cells without significant toxic effects on normal cells and is one of the most powerful biologically active factors for direct tumor killing so far [45].

**Figure 6:**
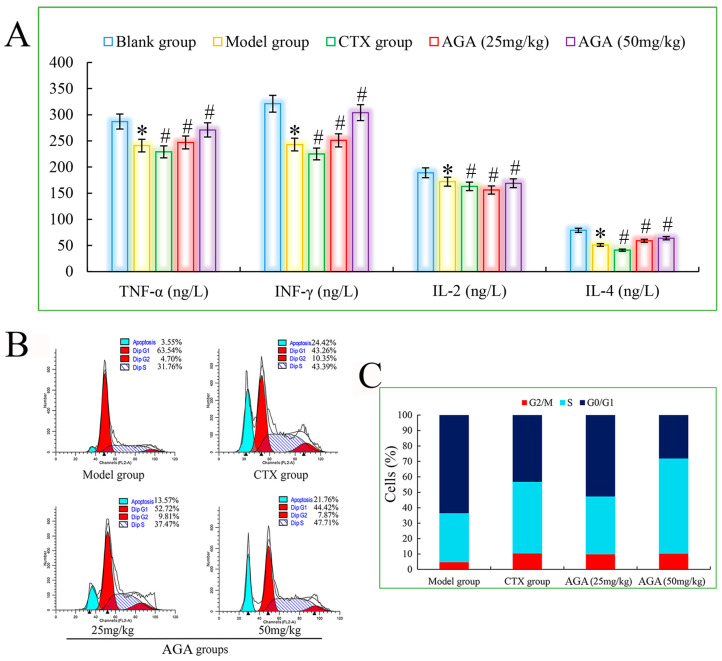
The blood cytokines detection of H22 tumor-bearing mice (**A**). Cell cycle phase distribution of H22 tumor-bearing mice, histograms of DNA content in the cells (**B**) and bar graph of cell proportion in G0/G1, S and G2/M phases (**C**). * *p* < 0.05, compared with Blank group; ^#^ *p* < 0.05, compared with Model group.

IL-2 has an important role in the immune response of the body and antiviral infection, etc. IL-4 has immunomodulatory effects on B cells, T cells, mast cells, macrophages and hematopoietic cells. IFN-γ is a highly potent antiviral bioactive substance with broad immunomodulatory effects. Therefore, assaying the biological activity of cytokines is an important step in exploring the anti-tumor immune effects induced by AGA in H22 tumor-bearing mice [46]. As shown in Figure 6A, the levels of TNF-α, IL-2, IL-4 and IFN-γ in the model group were significantly decreased compared with the blank group (*p* < 0.05), and the levels of all cytokines in the dose groups were significantly increased compared with the model group (*p* < 0.05) in a dose-dependent manner. At the same time, the mice in the CTX group had the lowest cytokine levels, suggesting that CTX exerts some toxic side effects on the immune system of the mice. All the above results suggest that AGA can inhibit the proliferation of H22 tumor cells and promote the secretion of various cytokines in vivo to improve the body immunity [47].

### 3.6. Cell Cycle Distribution of Solid Tumor

The cell cycle responds to the rate of cell proliferation and plays an important role in the diagnosis and prognosis of tumors. A complete cell cycle consists of pre-DNA synthesis (G1 phase), DNA synthesis (S phase), and DNA synthesis (G2 phase), so we select PI fluorescent dyes that bind to DNA and analyze the cell cycle by measuring the amount of DNA [48,49]. It can be seen from Figure 6B,C) that the apoptosis rate of the model group was only 3.55%, indicating that the H22 cells in the model group were basically in a normal growth state. After AGA treatment, the apoptosis rate increased to 13.57% and 21.76%, while the apoptosis rate of the CTX group reached to 24.42%. The proportion of S phase increased from 31.76% in model group to 37.47% (25 mg/mL) and 47.71% (50 mg/mL) in dose groups, suggesting that AGA can induce apoptosis of H22 cells by arresting apoptosis in S phase [50].

## 4. Conclusions

In this paper, acetylaminoglucose AGA was obtained by heating crude chitin dissolved in concentrated hydrochloric acid, ethanol precipitation and purification by Sephadex G25. The purity and characteristic absorption peaks of AGA were also detected by UV, HPLC and FT-IR spectroscopy, and the anti-tumor activity of AGA was investigated by establishing an animal model of H22. The results of animal experiments showed that AGA could induce apoptosis of H22 tumor cells in vivo by improving the status of immune organs, increasing the phagocytic ability of peritoneal macrophages, the killing power of NK cells and the proliferation ability of lymphocytes, increasing the levels of cytokines (TNF-α, IL-2, IL-4 and IFN-γ), and regulating the distribution of lymphocyte subpopulations, thus achieving anti-tumor effects in vivo. These findings will provide the basis for the use of AGA as an anti-tumor agent.

## Data Availability

Not applicable.
